# Supplementary material for: Flour Functionality, Nutritional Composition, and In Vitro Protein Digestibility of Wheat Cookies Enriched with Decolourised Moringa oleifera Leaf Powder
Source: Foods. 2024 May 25;13(11):1654. doi: 10.3390/foods13111654 (PMC11171687; doi:10.3390/foods13111654)
Supplement: Supplementary file 1 [file foods-13-01654-s001.zip › foods-3003917-supplementary.pdf]

Table S1

Retention time, linear calibration range and R^2 for amino and phenolic acids determination in wheat-Moringa cookies

Amino Acids	Retention time	Linear range	R^2
Alanine	3.7	9.7 - 970	0.9987
Arginine	3.6	51.5 - 1030	0.9919
Asparagine	3.6	9.3 - 930	0.9940
Aspartic acid	3.7	25.8 - 1030	0.9993
Cysteine		N/D	
Glutamic acid	3.9	25.8 - 1030	0.9985
Glutamine	3.75	24.8 - 990	0.9995
Glycine	3.5	25.5 - 1020	0.9981
Histidine	3.5	24 - 960	0.9990
4-Hydroxy-L-proline	3.8	25 - 1000	0.9995
Isoleucine	6.8	25 - 1000	0.9992
Leucine	7.0	9.8 - 980	0.9998
Lysine	3.3	24.3 - 970	0.9983
Methionine	5.9	10.3 - 1030	0.9992
Phenylalanine	7.9	10.1 - 1010	0.9995
Proline	4.6	9.9 - 990	0.9989
Serine	3.6	9.7 - 970	0.9993
Threonine	3.8	10.5 - 1050	0.9987
Tryptophan	8.6	9.9 - 990	0.9968
Tyrosine	6.6	37.5 - 750	0.9847
Valine	5.7	52.5 - 1050	0.9977
**Phenolic Acids**			
Fumaric acid	7.96	50-1000	0.9996
Gallic acid	8.29	50-1000	0.9989
Chlorogenic acid	9.75	50-1000	0.9980
Caffeic acid	10.53	10-1000	0.9996
Syringic acid	10.68	400-1000	0.9128
Sinnapic acid	11.25	50-800	0.9987
p-Coumaric acid	11.33	50-1000	0.9995
Ferullic acid	11.38	50-800	0.9899
